# Low levels of Caspase-3 predict favourable response to 5FU-based chemotherapy in advanced colorectal cancer: Caspase-3 inhibition as a therapeutic approach

**DOI:** 10.1038/cddis.2016.7

**Published:** 2016-02-04

**Authors:** L Flanagan, M Meyer, J Fay, S Curry, O Bacon, H Duessmann, K John, K C Boland, D A McNamara, E W Kay, H Bantel, H Schulze-Bergkamen, J H M Prehn

**Affiliations:** 1 Department of Physiology and Medical Physics, Royal College of Surgeons in Ireland, Dublin, Ireland; 2 Centre for Systems Medicine, Royal College of Surgeons in Ireland, Dublin, Ireland; 3 Department of Pathology, Beaumont Hospital, Dublin, Ireland; 4 Department of Surgery, Beaumont Hospital, Dublin, Ireland; 5 Department of Gastroenterology, Hepatology and Endocrinology, Hannover Medical School, Hannover, Germany; 6 Department of Medical Oncology, National Center for Tumor Diseases, Heidelberg, Germany; 7 Department of Gastroenterology and Oncology, Marien-Hospital Wesel, Wesel, Germany

## Abstract

Colorectal cancer (CRC) is one of the most common cancers in the Western world. 5-Fluorouracil (5FU)-based chemotherapy (CT) remains the mainstay treatment of CRC in the advanced setting, and activates executioner caspases in target cells. Executioner caspases are key proteins involved in cell disassembly during apoptosis. Activation of executioner caspases also has a role in tissue regeneration and repopulation by stimulating signal transduction and cell proliferation in neighbouring, non-apoptotic cells as reported recently. Tissue microarrays (TMAs) consisting of tumour tissue from 93 stage II and III colon cancer patients were analysed by immunohistochemistry. Surprisingly, patients with low levels of active Caspase-3 had an increased disease-free survival time. This was particularly pronounced in patients who received 5FU-based adjuvant CT. In line with this observation, lower serum levels of active Caspase-3 were found in patients with metastasised CRC who revealed stable disease or tumour regression compared with those with disease progression. The role of Caspase-3 in treatment responses was explored further in primary human tumour explant cultures from fresh patient tumour tissue. Exposure of explant cultures to 5FU-based CT increased the percentage of cells positive for active Caspase-3 and Terminal Deoxynucleotidyl Transferase dUTP Nick end Labelling (TUNEL), but also the expression of regeneration and proliferation markers *β*-Catenin and Ki-67, as well as cyclooxygenase-2 (COX-2). Of note, selective inhibition of Caspase-3 with Ac-DNLD-CHO, a selective, reversible inhibitor of Caspase-3, significantly reduced the expression of proliferation markers as well as COX-2. Inhibition of COX-2 with aspirin or celecoxib did not affect Caspase-3 levels but also reduced Ki-67 and *β*-Catenin levels, suggesting that Caspase-3 acted via COX-2 to stimulate cell proliferation and tissue regeneration. This indicates that low levels of active Caspase-3 may represent a new predictor of CT responsiveness, and inhibition of Caspase-3, or antagonising downstream effectors of Caspase-3 paracrine signalling, such as COX-2 may improve patient outcomes following CT in advanced CRC.

Colorectal cancer (CRC) has the third highest incidence rate and the fourth highest mortality rate of any cancer in the Western world. Surgery alone, or combined with 5-Fluorouracil (5FU)-based chemotherapies, is the current treatment option for stage II and advanced, stage III CRC. The response rate to palliative 5FU-based treatments for metastatic stage IV CRC is *c*. 40–50%, but median overall survival (OS) remains low at *c.*16–19 months.

Most common chemotherapeutics lead to the induction of apoptosis in the targeted cancer cell. Caspases are cysteine proteases that are crucial to the morphological and biochemical changes that occur during apoptosis. Caspases-3 and -7 are the main executioner caspases in the cell. Caspase-3 targets structural substrates leading to cell disassembly and DNA fragmentation.^1^ Caspase-7 has similar functions and in the absence of Caspase-3 apoptosis will still occur, albeit with a slower kinetic.^2^ Despite sharing many substrates and compensating for each other in apoptosis, there are specific functions of Caspase-3 that cannot be replaced by Caspase-7, as Caspase-3 acts on a wider range of substrates.^3^ Caspase-3 has also been shown to be involved in a number of processes that are not directly linked to cell disassembly. Activation of executioner caspases during apoptosis has an important role as a negative regulator of the immune response by triggering the release of anti-inflammatory cytokines and suppressing the inflammatory response.^4, 5^ Alongside these roles in immune suppression, executioner caspases have a role in tissue regeneration and proliferation. Although Caspase-3 activation causes cell death in the host cell, it has been found to stimulate cell proliferation in neighbouring, non-apoptotic cells. This phenomenon is important in wound healing and tissue regeneration,^6, 7^ but may potentially hinder success in chemotherapy (CT) regimens. Although less resistant cells may activate Caspase-3 and undergo apoptosis, these dying cells may send out signals to more resistant cancer cells promoting proliferation and repopulation. In many cancer types, patients with poor prognosis display highly proliferating tumours.^8, 9^

It has been demonstrated in a model of head regeneration in *hydra* that caspases are required for nuclear translocation of *β*-catenin and Wnt signalling during regeneration, linking for the first time caspase activation mechanistically with tissue regeneration.^10^ Cyclooxygenase-2 (COX-2) activation may link these two processes: It is known that Wnt activation in cancer tissue and cancer stem cells is activated by Prostaglandin E2 (PGE2), which is produced predominantly from arachidonic acid (AA) by COX and Prostaglandine E synthases.^11^ Zhao *et al.*^12^ showed that Caspase-3 generates a truncated, active form of calcium-independent Phospholipase A_2_, iPLA2 (514–806). Activation of iPLA2 (514–806) generates lysophosphatide acid and arachidonic acid, a substrate for COX-2. In a model of breast cancer, Huang *et al.*^13^ showed an increase in PGE2 levels and cell proliferation downstream of Caspase-3 activation upon apoptosis induction, and that there was reduced AA release, PGE2 production and cell proliferation in Casp3−/− cells which could be rescued by overexpression of iPLA2 (514–806). Similar results have been reported in a model of skin regeneration, where the authors demonstrated that Caspase-3 was required to stimulate proliferation and regeneration via Caspase-3-dependent iPLA2 activation, resulting in AA synthesis and subsequent PGE2 and Wnt/beta-catenin signalling.^14^

In the present study, we therefore examined Caspase-3 as a prognostic marker in stage 2/3 as well as in metastatic CRC patients, and further explored the role of caspase-3 as a potential predictive biomarker for 5FU-based CT and therapeutic target in CRC using primary human tumour explant culture.

## Results

### Low Caspase-3 activation indicates increased disease-free survival and better outcome for CRC patients

**Figure 1:**
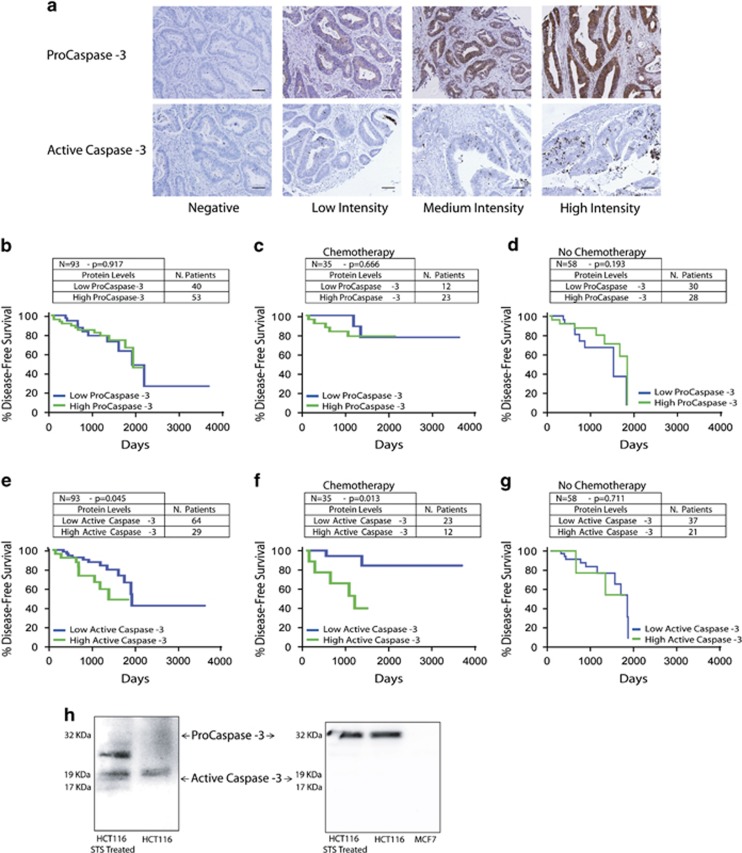
Low active Caspase-3 levels in patient tumours results in a significantly better disease-free survival time compared with high active Caspase-3 levels in stage 2/3 CRC patients receiving 5FU-based CT. (**a**) TMA sections, comprises 93 patient tumours, were stained for Pro- and active Caspase-3 and scored based on extent and intensity of staining (scale bar=100 *μ*m). (**b**) Protein levels were correlated to patient outcome, and patients with low ProCaspase-3 (blue) did similarly to those with high ProCaspase-3 (green). (*N*=93, 40 patients expressed low protein levels and 53 patients expressed high levels). (**c** and **d**) This survival pattern was observed whether patients received CT (**c**; *N*=35, 12 patients expressed low protein levels, with 2 experiencing recurrence, and 23 patients expressed high levels, with 5 experiencing recurrence) or not (**d**; *N*=58, 30 patients expressed low protein levels, with 8 experiencing recurrence, and 28 patients expressed high levels, with 7 experiencing recurrence). (**e**) Patients with low levels of active Caspase-3 were found to have a significantly better disease-free survival than patients with high Caspase-3 (*P*=0.045; *N*=93, 64 patients expressed low protein levels and 29 patients expressed high levels). (**f**) In the CT group, this effect was further emphasised and disease-free survival was again increased in patients with low active Caspase-3 (*P*=0.013; *N*=35, 23 patients expressed low protein levels, with 2 experiencing recurrence, and 12 patients expressed high levels, with 5 experiencing recurrence). (**g**) In the patients who received no chemotherapy this effect on disease-free survival was lost (*N*=58, 37 patients expressed low protein levels, with 11 experiencing recurrence, and 21 patients expressed high levels, with 3 experiencing recurrence). (**h**) Western blot analysis on HCT116 cell line, untreated and treated with STS, confirmed that the antibodies (cleaved Caspase-3 antibody, 9661, CST and ProCaspase-3 Antibody, HPA002643, Atlas Antibodies) only detected the active (cleaved) form of Caspase-3 and the proform, respectively. MCF7 cells were included as a negative control for specificity determination of the ProCaspase-3 antibody

To explore the association of Caspase-3 with patient outcome, we examined tissue microarrays (TMAs) consisting of tumour tissue from a total of 93 CRC patients. This cohort consisted of stage II and III patients who underwent surgery followed by observation alone (*N*=58), or observation alongside 5FU-based CT (*N*=35). Slides were stained for both Pro- and active Caspase-3 (Figure 1a).

Tumour areas were defined as positively stained cell structures within the overall malignant epithelium of the colonic crypt. Tumours were scored semi-quantitatively using an own adaptation from Allred score technique, on a scale of 0–3 based on the extent and intensity of staining (0=no stained cells; 1=low. Faint, partial to moderate staining in ≤20% of cells; 2=medium. Weak, partial to moderate staining in >20% of cells; 3=high. Strong, moderate to complete staining in ≥70% of cells). Following the recommendations made in the American Society of Clinical Oncology (ASCO)–College of American Pathologists (CAP) Test Guideline,^15^ a cutoff value of 1% of stained cells was used to discriminate between positive (>1%) and negative (<1%) cases.^16^ Patients were allocated as having either a low or high level of Caspase-3 and active Caspase-3 based on the median level of Caspase-3 in all patients. Survival analysis of the total cohort demonstrated that ProCaspase-3 levels were not associated with survival time (Figure 1b, *P*=0.917; *N*=93, 40 patients expressed low protein levels, of which 10 recurred, and 53 patients expressed high levels, of which 13 recurred). However, patients who had low levels of active Caspase-3 had a significantly improved disease-free survival (DFS) when compared with patients with high levels of the active protein (Figure 1e, *P*=0.045; *N*=93, 64 patients expressed low protein levels, of which 15 recurred, and 29 patients expressed high levels, of which 6 recurred). When we examined active Caspase-3 levels in the cohort of patients who received CT we found that again patients who had low levels of active Caspase-3 had a significantly improved DFS in comparison with patients with high levels of the active protein (Figure 1f, *P*=0.013; *N*=35, 23 patients expressed low protein levels, with 2 experiencing recurrence, and 12 patients expressed high levels, with 5 experiencing recurrence). In the group of patients who did not receive CT active Caspase-3 levels could not stratify patients (Figure 1g, *P*=0.711; *N*=58, 37 patients expressed low protein levels, with 11 experiencing recurrence, and 21 patients expressed high levels, with 3 experiencing recurrence). Similarly, low ProCaspase-3 levels showed no association with DFS whether the patients received CT (Figure 1c, *P*=0.666; *N*=35, 12 patients expressed low protein levels, with 2 experiencing recurrence, and 23 patients expressed high levels, with 5 experiencing recurrence) or not (Figure 1d, *P*=0.193; *N*=58, 30 patients expressed low protein levels, with 8 experiencing recurrence, and 28 patients expressed high levels, with 7 experiencing recurrence). These data suggested active Caspase-3 in tumour tissue as a predictive marker for CRC patients receiving 5FU-based CT, allowing for the identification of patients who are likely to respond to therapy.

Western blot analysis performed on staurosporine-treated human colon cancer HCT116 cells confirmed that the ProCaspase-3 and the active Caspase-3 antibodies used only detected the pro-enzyme and cleaved form, respectively (Figure 1h).

To examine a potential correlation between the expression of active caspase-3 and proliferation markers, we also evaluated Ki-67 levels in the TMAs. Semi-quantitative analysis of Ki-67 staining and active Caspase-3 staining on the CRC TMAs did not reveal a significant correlation (Spearman correlation analysis: rho=0.007, *P*=0.95); however, there was a positive correlation in the CT *versus* untreated group (rho=0.10, *P*=0.50 *versus* rho=−0.03, *P*=0.82) albeit none of these correlations were statistically significant. Survival analysis demonstrated that Ki-67 levels at time of tumour resection were not associated with length of survival (Supplementary Figure).

### Caspase-3 activity in the serum of metastatic CRC patients is negatively associated with patient response to therapy

**Figure 2:**
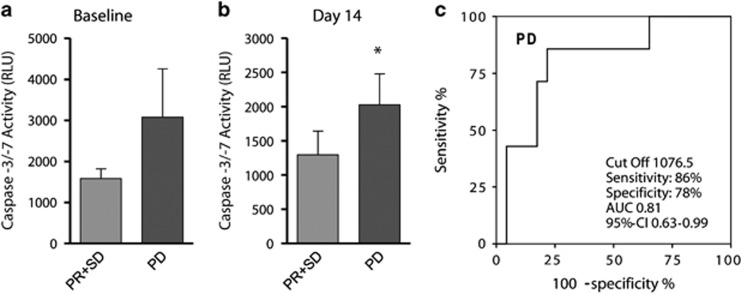
High levels of Caspase-3/-7 activity in the serum are indicative of PD in metastasised colorectal carcinoma patients (**a**). In a cohort of 30 metastatic CRC patients, Caspase-3/-7 activity levels in the serum were evaluated at baseline (before therapy) according to their treatment response following the first cycle of CT. PD (*N*=7) was associated with higher Caspase-3/-7 activity compared with SD (*N*=14) and partial response (PR; *N*=9). (**b**) Patients who displayed PD revealed significantly higher levels of Caspase-3/-7 activity in their serum when compared with patients who displayed SD or partial tumour regression at day 14 following commencement of therapy. (**c**) Caspase-3/-7 activity levels in these patients could predict PD at day 14 with a sensitivity of 86% and a specificity of 78%. *P<0.05

To validate our findings of Caspase-3 as a prognostic biomarker using a methodologically different approach, we examined whether Caspase-3/-7 activity in the serum may also correspond to response to therapy in metastatic CRC patients. Patient cohort consisted of 30 metastatic CRC patients who received a standard CT, that is, FOLFOX or FOLFIRI, with or without anti-EGFR /-VEGFR antibody treatment (Table 2). Baseline levels of Caspase-3/-7 activity in serum were higher in patients who went on to have progressive disease (PD) and a poor response to therapy (*n*=7, 3082.7±1173.1 relative light units (RLU)), while those patients who showed a response to therapy and stable disease (SD, *n*=14) or partial tumour regression (PR, *n*=9) displayed lower Caspase-3/-7 activity (1579.8±239.8 RLU; Figure 2a). This effect was further emphasised following 14 days of treatment, when Caspase-3/-7 activity was significantly (*P*<0.05) higher in patients with PD (2029.3±450.0 RLU) compared with SD and PR patients (1295.2±349.5 RLU; Figure 2b). We then calculated the cutoff value for Caspase-3/-7 activity, which allows the prediction of disease progression with the best compromise sensitivity/specificity. As demonstrated by receiver operating characteristics (ROC) analysis (Figure 2c), we identified a cutoff value of 1076.5 RLU for Caspase-3/-7 activity in the serum which could early (day 14 of therapy) predict PD with a sensitivity of 86% and a specificity of 78% (area under the curve (AUC) 0.81, 95% confidence interval (CI) 0.63–0.99).

### 5FU-based CT activates Caspase-3 and increases proliferation markers in patient-derived primary human tumour explant cultures

**Figure 3:**
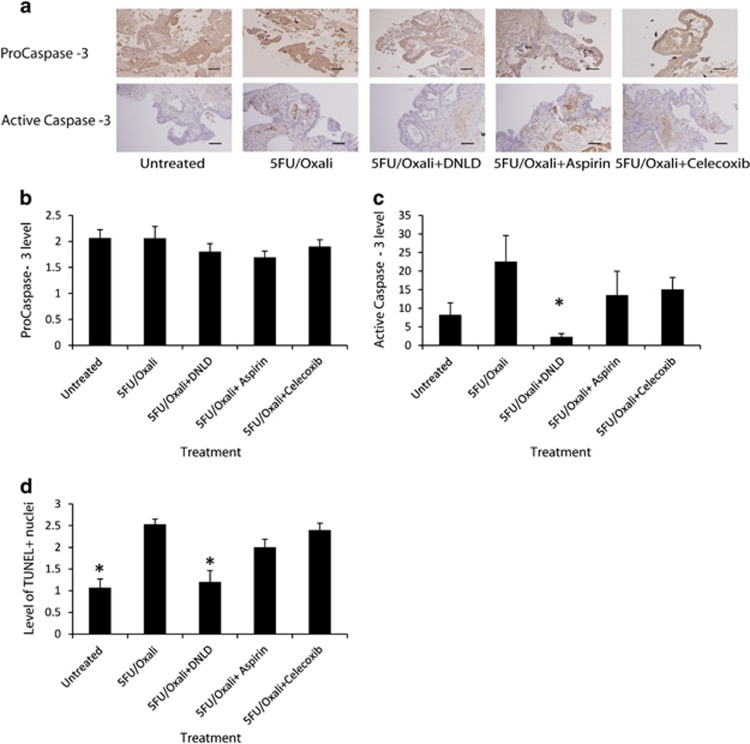
Caspase-3 is activated and apoptosis is induced following CT, and COX-2 inhibitors do not affect this process. (**a**) Fresh patient colon tumour tissue (*N*=19 patients) was treated with 5FU-based CT alone and in combination with specific Caspase-3 inhibitor (DNLD) and COX-2 inhibitors (Aspirin and Celecoxib). Tissue was sectioned and stained for ProCaspase-3 and active Caspase-3 (**a**) and scored based on extent and intensity of staining (scale bar=100 *μ*m). (**b** and **c**) Levels of ProCaspase-3 (**b**) and active Caspase-3 (**c**) were quantified. ProCaspase-3 levels were unaffected by treatment regimes, while active Caspase-3 was significantly reduced (5FU/Oxali *versus* 5FU/Oxali+DNLD, *P*=0.021) by specific Caspase-3 inhibition alongside 5FU-based CT but unaffected by other treatments. (**d**) Using a TUNEL-based assay, levels of apoptosis were examined. Chemotherapy-induced apoptosis (Untreated *versus* 5FU/Oxali*, P*=0.00013), which was reduced with Caspase-3 inhibition (5FU/Oxali *versus* 5FU/Oxali+DNLD, *P*=0.001). COX-2 inhibition did not reduce apoptosis induction. *Denotes statistically significant difference, *P*-values⩽0.05.

To investigate in more detail whether CT-induced Caspase-3 activation may drive proliferation, we used a novel explant technique that utilises haemostatic gelatine dental sponges as a support, upon which fresh patient tissue can be placed and cultured.^17, 18^ Treatments were added to the media surrounding the sponge and diffused through the sponge to be taken up by the tissue. Using this model, we treated 19 fresh colon tumours taken at surgery with 5FU-based CT (30 *μ*g 5FU/10 *μ*g Oxaliplatin) alone and in combination with a specific Caspase-3 inhibitor, Ac-DLND-CHO^19^ (30 *μ*g 5FU/10 *μ*g Oxali+200 nM Ac-DNLD-CHO; hereafter DLND) and COX-2 inhibitors Aspirin and Celecoxib (30 *μ*g 5FU/10 *μ*g Oxali+2.5 mM Aspirin and 30 *μ*g 5FU/10 *μ*g Oxali+50 *μ*M Celecoxib). Tissues were then fixed, embedded, sectioned, and stained for Pro- and active Caspase-3 (Figure 3a). ProCaspase-3 levels were unchanged across the treatment groups (Figure 3b); however, active Caspase-3 levels increased following 5FU-based CT. Specific inhibition of Caspase-3 in combination with 5FU-based treatment effectively inhibited Caspase-3 activation as expected (5FU/Oxali *versus* 5FU/Oxali+DNLD; *P*=0.021; analysis of variance (ANOVA) and *post hoc* Tukey's test). To examine the proposed Caspase-3-dependent proliferation pathway COX-2 inhibitors, aspirin and celecoxib, were utilised and found to not be effective in inhibiting Caspase-3 activation (Figure 3c). Apoptosis induction in tumours was also verified with a Terminal Deoxynucleotidyl Transferase dUTP Nick end Labelling (TUNEL)-based apoptosis assay. The results mirrored the results of Caspase-3 activation, whereby apoptosis was induced following 5FU-based CT (Untreated *versus* 5FU/Oxali; *P*=0.00013; ANOVA and *post hoc* Tukey) but Caspase-3 inhibition reduced apoptosis (5FU/Oxali *versus* 5FU/Oxali+DNLD; *P*=0.001; ANOVA and *post hoc* Tukey). COX-2 inhibition had no significant effect on apoptosis induction (Figure 3d). These results first validate our explant model and show that treatments reached the tissue, and tissue responded to each treatment regime. Furthermore, the results show that COX-2 inhibitors did not affect Caspase-3 activation and apoptosis induction.

### Inhibition of Caspase-3 and COX-2 results in a reduction in the expression of proliferation markers

**Figure 4:**
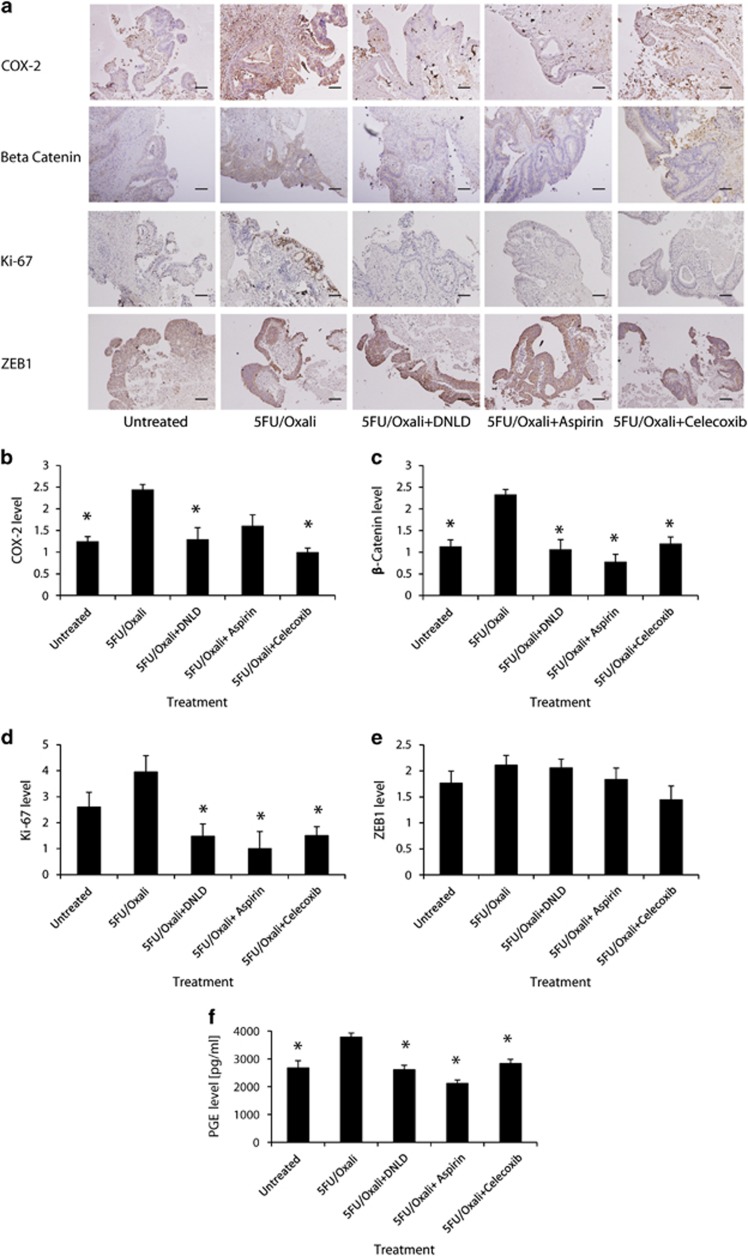
Chemotherapy-induced Caspase-3 activation increases expression of proliferation markers, and inhibition of Caspase-3 and its downstream effectors antagonises this proliferation signature. (**a**) Fresh patient colon tumour tissue was treated with 5FU-based CT alone and in combination with specific Caspase-3 inhibitor (DNLD) and COX-2 inhibitors (Aspirin and Celecoxib). Tissue was sectioned and stained for COX-2, *β*-Catenin, Ki-67, and ZEB1 (**a**) and scored based on the extent and intensity of staining (scale bar=100 *μ*m). (**b**) COX-2 levels were significantly increased following CT when compared with untreated tissue (*P*=0.00017). Expression of COX-2 was reduced when Caspase-3 was inhibited during CT (*P*=0.000443) and celecoxib co-treatment also significantly antagonised COX-2 expression (*P*=0.000242). (**c**) CT significantly increased *β-*Catenin levels when compared with untreated tissue (*P*=0.00237). Specific Caspase-3 inhibition reduced *β*-Catenin expression significantly (*P*=0.001), and inhibition of downstream Caspase-3 effectors via aspirin and celecoxib resulted in reduction in *β*-Catenin expression (*P*=0.0003 and *P*=0.003, respectively). (**d**) Expression of Ki-67 was not significantly increased following CT when compared with untreated tissue. Inhibition of Caspase-3 in concert with CT (*P*=0.008) and its downstream effectors alongside 5FU-based CT reduced Ki-67 expression (5FU/Oxali *versus* 5FU/Oxali+Celecoxib, *P*=0.021, 5FU/Oxali *versus* 5FU/Oxali+Aspirin, *P*=0.003). (**e**) Expression of ZEB1 was not altered across treatment groups, suggesting that the EMT does not accompany Caspase-3-driven proliferation. (**f**) 5FU-based regimen caused a significant increase in PGE2 release (Untreated *versus* 5FU/Oxali, *P*=0.034), while specific Caspase-3 and COX-2 inhibition reduced PGE2 media levels (5FU/Oxali *versus* 5FU/Oxali+DNLD, *P*=0.033; 5FU/Oxali *versus* 5FU/Oxali+Aspirin, *P*=0.021; 5FU/Oxali *versus* 5FU/Oxali+Celecoxib, *P*=0.045; *n*=4–6 explants evaluated per treatment; ANOVA and *post hoc* Tukey). *Denotes statistically significant difference, *P*-values⩽0.05.

**Figure 5:**
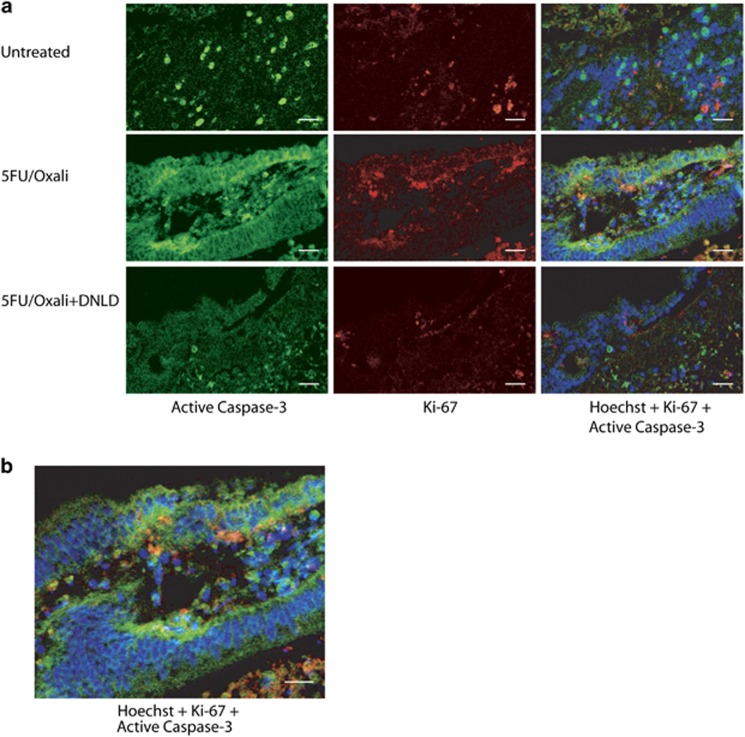
Active Caspase-3 and Ki-67 do not colocalise. (**a**) Tumour explant cultures remained untreated or were treated with 5FU/Oxali alone or in combination with the specific Caspase-3 inhibitor, DNLD. Tissues were sectioned and double stained for immunofluorescence for active Caspase-3 (green, left panels) and Ki-67 (red, middle panels). Nuclei were stained with Hoechst (blue, overlay in right panels). Represented images from *n*=x–y explant cultures per treatment condition are shown (scale bar=50 *μ*m). (**b**) Detail of active Caspase-3 and Ki-67 staining in a 5FU/Oxaliplatin-treated culture, showing that Ki-67 nuclear staining corresponded to the zone of proliferating epithelial progenitors of the colonic crypts, where distinct stem cell, proliferating, and differentiating compartments are localised (scale bar=50 *μ*m)

Once we had established that our primary tumour explant culture system allowed for efficient treatment of tumour tissue, we next examined by immunohistochemistry how our treatment scenarios altered proliferation profiles in tumours. We analysed COX-2, *β*-Catenin, and Ki-67 (Figure 4a). As previously mentioned, COX-2 is activated in the proposed Caspase-3-dependent proliferation pathway^17^ leading to *β*-Catenin translocation, culminating in proliferation in neighbouring cells. Ki-67 was also analysed due to its preferential expression in all active stages of the cell cycle.^20, 21^ Chemotherapy caused COX-2 and *β*-Catenin expression to increase (Figures 4b and c), and there was a significant difference between levels of these proteins in untreated *versus* 5FU/Oxali-treated tissue (COX-2; Untreated *versus* 5FU/Oxali, *P*=0.00017, *β*-Catenin; Untreated *versus* 5FU/Oxali, *P*=0.00237; ANOVA and *post hoc* Tukey). Interestingly, Caspase-3 inhibition in combination with CT (30 *μ*g 5FU/10 *μ*g Oxali+200 nM DNLD) significantly reduced the expression of *β*-Catenin and Ki-67 when compared with CT alone (Figures 4c and d; *β*-Catenin; 5FU/Oxali *versus* 5FU/Oxali+DNLD; *P*=0.001; Ki-67; 5FU/Oxali *versus* 5FU/Oxali+DNLD; *P*=0.008; ANOVA and *post hoc* Tukey). Furthermore, Caspase-3 inhibition in combination with CT also reduced COX-2 levels (Figure 4b; COX-2; 5FU/Oxali *versus* 5FU/Oxali+DNLD; *P*=0.000443; ANOVA and *post hoc* Tukey). Chemotherapy also caused a significant increase in PGE2 release into the media (Figure 4f, Untreated *versus* 5FU/Oxali; *P*=0.034), while specific Caspase-3 and COX-2 inhibition reduced PGE2 release (Figure 5f, 5FU/Oxali *versus* 5FU/Oxali+DNLD, *P*=0.033; 5FU/Oxali *versus* 5FU/Oxali+Aspirin, *P*=0.021; 5FU/Oxali *versus* 5FU/Oxali+Celecoxib, *P*=0.045; *n*=4–6 explants per condition; ANOVA and *post hoc* Tukey).

Celecoxib in combination with CT (30 *μ*g 5FU/10 *μ*g Oxali+50 *μ*M celecoxib) also significantly reduced the proliferation signature when compared with CT alone (Figures 4b and d; COX-2; 5FU/Oxali *versus* 5FU/Oxali+Celecoxib; *P*=0.000242; *β*-Catenin; 5FU/Oxali *versus* 5FU/Oxali+Celecoxib; *P*=0.00201; Ki-67; 5FU/Oxali *versus* 5FU/Oxali+Celecoxib; *P*=0.021; ANOVA and *post hoc* Tukey). Aspirin in combination with CT (30 *μ*g 5FU/10 *μ*g Oxali+2.5 mM Aspirin) efficiently reduced expression of *β*-Catenin and Ki-67 (Figures 4c and d; *β*-Catenin; 5FU/Oxali *versus* 5FU/Oxali+Aspirin; *P*=0.0003; Ki-67; 5FU/Oxali *versus* 5FU/Oxali+Aspirin; *P*=0.003; ANOVA and *post hoc* Tukey). Aspirin co-treatment did not significantly reduce expression of COX-2.

Epithelial mesenchymal transition (EMT) is a process that occurs in several cancer types leading to increased motility and invasiveness of cancer cells. Consequently, tumours with high EMT often have a poor prognosis.^22^ The Zinc Finger E-Box Binding Homeobox (ZEB) family of transcription factors is integral to EMT.^23^ ZEB1 was previously shown to supress E-Cadherin expression via PGE_2_-mediated signalling.^24^ Therefore, we investigated whether Caspase-3-driven proliferation might impact EMT (Figure 4e). Explant samples were examined for ZEB1 expression. Expression was not altered across treatment groups, suggesting that the EMT does not accompany Caspase-3-driven proliferation.

We finally performed double immunofluorescence staining for active Caspase-3 and Ki-67 to obtain spatial information regarding cell death *versus* cell proliferation in the same tissue section. Semi-quantitative analysis of seven explant cultures per treatment showed that the pattern reported in the immunofluorescence staining was similar to that obtained by immunohistochemistry (data not shown). Immunoreactivity was present in both untreated and 5FU/Oxali-treated explant cultures, with higher staining in 5FU/Oxali-treated cultures (Figure 5a). Explant cultures treated with 5FU/Oxali+DLND showed reduced levels of active Caspase-3 and Ki-67 (Figure 5a). Expression areas in 5FU/Oxali-treated cultures did not show overlap, suggesting that the population of cells with high proliferation differed from those with high caspase-3 activity. Active Caspase-3 staining was predominantly located in the cytoplasm of epithelial cells of colonic crypts (Figure 5b). Ki-67 nuclear staining corresponded to the zone of proliferating epithelial progenitors of the colonic crypts, where distinct stem cell, proliferating, and differentiating compartments are localised.^25^

## Discussion

Executioner caspases such as Caspase-3 and Caspase-7 have long been recognised as the key proteases involved in cellular degradation during apoptosis. Interestingly, in recent years there is much evidence supporting a role for Caspase-3 in paracrine signalling, which may also influence signal transduction and gene expression changes in neighbouring cells that themselves did not activate caspases.^6, 26, 27^ Analysis of TMAs of stage 2 and 3 CRC tumours revealed that low levels of active Caspase-3 conferred a significant survival advantage. We found that in patients receiving CT, active Caspase-3 levels gave an even more notable survival advantage, suggesting that while CT is activating Caspase-3, high levels of the active protein may contribute to the eventual relapse. In patients receiving CT, levels of low ProCaspase-3 were suggestive of improved DFS for patients, although not significant, implying that ProCaspase-3 levels may be determinant of active Caspase-3 levels. In patients who did not receive CT, Caspase-3 levels were not associated with DFS, indicating that this phenomenon is present only when CT is administered. This indicates that active Caspase-3 levels represent a potential predictive biomarker of patient outcome and response to 5FU-based CT, helping to identify individuals likely to respond to CT.

We also examined Caspase-3/-7 activity in the serum of 30 metastatic CRC patients. This analysis revealed that serum Caspase-3/-7 activity was highest in patients with PD status, and the poorest outlook, while those patients who respond to therapy with either PD or SD have significantly lower levels of activity. This indicated that circulating levels of active Caspase-3/-7 influence disease progression and high levels were similarly indicative of PD in the metastatic setting where there is a higher tumour load. Cytokeratin 18 (CK18) is cleaved by caspases during apoptosis and released into the bloodstream.^16, 28^ It has been recently reported that in the serum of SCCHN patients levels of CK18 and cleaved Caspase-3 were increased compared with that of normal healthy controls. High CK18 levels and high Caspase-3 activity were also associated with shorter disease-free progression in these patients.^29^ Taken together, these data present evidence pointing to Caspase-3 as a potential predictive biomarker of response to 5FU-based CT in advanced CRC. Measurement of Caspase-3 in the tumour or of Caspase-3/-7 activity in the serum may enable reliable stratification of patients based on response and allow for the identification of patients likely to respond poorly to 5FU-based CT. However, analysis in larger cohorts and long-term studies are required to further validate Caspase-3 or Caspase-3/-7 activity as a biomarker for early prediction of treatment outcome or disease progression in CRC patients.

Because genotoxic therapy activates Caspase-3 to induce tumour cell death, it may appear contradictory that low levels of caspase-3 may be beneficial for responses to genotoxic CT. However, colorectal tumours should be considered to be highly heterogeneous with subpopulations of cells that vary in their responses to CT. Moreover, each cycle of therapy may only induce a fraction of tumour cells to undergo apoptosis (‘fractional kill hypothesis'). Hence, fractional tumour killing may induce caspase-dependent, compensatory cell proliferation in resistant cancer cells, leading to poor responsiveness to treatment or early patient relapse. Tumour growth rates are governed by the counterbalancing processes of cell proliferation and apoptosis. However the two events – Caspase-3 activation and cell proliferation – may occur at different time points and at different time scales, and may not be detectable *in situ*. Analysis of Ki-67 staining and active Caspase-3 staining on the CRC TMAs indicated a weak, positive correlation in the CT group, but no correlation in the untreated patient group, although none of these correlations were statistically significant. Limitations of this approach were the non-linearity of the scoring methodology and the fact that tumour staining was scored before CT. We therefore explored the relationship between Caspase-3 activation and cell proliferation in more detail in the controlled environment of tumour *ex vivo* cultures using a novel explant technique.^17, 18^ Previous studies employing gelatine dental sponges found that explant tumours retained tissue architecture and cellularity comparable to that observed in the original tumour section and retained similar expression of the key molecular markers.^17^ Using this method, we could examine Caspase-3 activation and individual patient response to CT and, furthermore, response to various antagonists alongside traditional CT. Our results demonstrated that Caspase-3 activity induces cell proliferation in patient tumours. Specific inhibition of Caspase-3 with Ac-DLND-CHO^19^ antagonised this proliferation, as did selective COX-2 inhibition with Celecoxib, suggesting that Caspase-3-dependent proliferation was mediated via COX-2. Double labelling of apoptosis activation (active Caspase-3 staining) and cell proliferation (Ki-67 staining) on CRC explant cultures demonstrated that areas of active Caspase-3 and Ki-67 staining in tumour cells did not overlap. Moreover, Ki-67 nuclear staining corresponded to the zone of proliferating epithelial progenitors of the colonic crypts, where distinct stem cell, proliferating, and differentiating compartments are localised.^25^ Interestingly, we also provide evidence that COX-2 levels were increased in response to 5FU/Oxali therapy. It has been demonstrated that COX-2 levels increase in a p53-dependent manner in response to genotoxic therapy,^30^ although p53 has also been shown to inhibit COX-2 gene induction.^31^ Our data suggest that COX-2 induction in a largely intact tumour microenvironment may also be Caspase-3 dependent, which adds further insight into the regulation of PGE2 production and *β*-catenin signalling during genotoxic stress in a more physiological setting. We also observed reduced COX-2 levels after Celecoxib treatment. This is in agreement with the reported positive feedback of prostaglandins including PGE2 on COX-2 induction.^32, 33^

Caspase-3 inhibition in concert with 5FU-based CT may be a viable approach for the treatment of advanced CRC. Clinical phase I/II trials using pan-caspase inhibitors are currently ongoing (http://ir.conatuspharma.com/releasedetail.cfm?releaseid=908153). One caveat of this approach may be that although specific caspase inhibition may impede tumour repopulation, it may lack sufficient cell-death induction to clear the initial tumour. However, genotoxic CT may activate both caspase-dependent and -independent cell-death pathways.^34^ Tumour growth in a lung cancer xenograft was significantly delayed by Caspase-3 inhibition, and tumours also displayed reduced vascularisation, highlighting the role of Caspase-3 in tumour growth and progression.^29^ Nevertheless, antagonising the repopulation pathway downstream of Caspase-3 may offer a more attractive alternative, allowing for cell-death induction but preventing proliferation signatures from being activated. With this in mind, we utilised the COX-2 inhibitors, aspirin and celecoxib, in our studies. Both treatments allowed for sufficient Caspase-3 activation, and displayed apoptosis induction. However, upon analysis of proliferation markers *β*-Catenin and Ki-67 we could demonstrate that antagonising this Caspase-3-dependent pathway at the COX-2 level reduced the expression of the proliferation signature in tumour tissue. Interestingly, regular use of aspirin after a diagnosis of CRC has been associated with a superior clinical outcome.^35^ There is also evidence suggesting that low daily doses of aspirin may be effective in reducing colorectal cancer risk.^36^ Taken together, these results suggest that COX-2 inhibitors may have potential as novel co-treatments to compliment traditional chemotherapies in advanced CRC.

## Materials and Methods

### TMA patient cohort

Patient tissue was collected and stored from the Departments of Surgery and Pathology, Beaumont Hospital, Dublin, Ireland. To ensure consistent quality and tumour presence, all tissues were microscopically evaluated by an experienced pathologist. Only tumour samples containing over 50% tumour cells were included in further analysis. Surgically resected colon tumour tissue from patients (*n*=93) was processed and formalin-fixed paraffin-embedded (FFPE) for construction of TMAs. Clinical follow-up, including gender, age at surgery, tumour grade, and adjuvant therapy regimes, was obtained through a review of medical records by a dedicated clinical research nurse (Table 1). Patients with hereditary forms of colorectal cancer were excluded. Informed consent was obtained from all patients; and ethical approval was obtained by the Beaumont Hospital Ethics Committee.

### TMA construction

Three 1-mm cores were taken from every case where available, using the Beecher Instruments arrayer (Sun Prairie, WI, USA) and placed into a paraffin block. Arrays consisted of 90–100 cores per array. Sections 5 *μ*m in thickness were cut, floated onto adhesive slides and baked overnight at 65 °C.

### Immunohistochemical staining

Antibodies were chosen based on their suitability for use on FFPE tissue and optimised. All staining was performed on a Leica Bond III automated immunostainer (Leica Microsystems, Wetzlar, Germany). The following primary antibodies were used: ProCaspase-3 (1 : 200; Rabbit polyclonal, HPA002643, Atlas Antibodies, Stockholm, Sweden), Active Caspase-3 (1:750; Rabbit polyclonal, 9661, CST, Cell Signaling Technology, Danvers, MA, USA), COX-2 (1 : 400; Mouse monoclonal; CAY160112-1, Cayman (@Cambridge Bioscience, Cambridge, UK)), and Ki-67 (MIB1) (1 : 100; Dako, Glostrup, Denmark, M7240). Negative controls for both rabbit and mouse antibodies (Negative Control Mouse Cocktail from mouse IgG1, IgG2a, IgG2b, IgG3, and IgM, IS750, Dako; Negative Control Rabbit Immunoglobulin fraction of serum from non-immunized rabbits, IS600, Dako) were included; and no staining was observed in these controls. Visualisation was performed using the enhanced diaminobenzidine tetrachloride with Harris′ haematoxylin as counterstain.

### Immunohistochemical scoring

Following staining, cores were scored based on the extent and intensity of the protein of interest. As multiple cores for each patient were arrayed, mean intensity scores were used for all subsequent analysis. Patients were allocated as those with either low or high protein levels, based on whether the score was above or below the median. The specificity of ProCaspase-3 and active Caspase-3 antibodies was confirmed by western blotting using the HCT116 cell line+STS treatment. MCF7 cells were included as a negative control.

### Serological analysis patient cohort

Patients with metastasised colorectal cancer (*n*=30) received a standard CT based on 5FU, leukovorin (LV) and oxaliplatin (FOLFOX) or 5FU, LV and irinotecan (FOLFIRI) +/− anti-EGFR or anti-VEGF antibody application. Sera were collected at baseline (before therapy) and day 14 of the first cycle of therapy and stored at −20 °C. Therapy response was assessed according to the Response Evaluation Criteria in Solid Tumours (RECIST) following the first cycle of therapy (Table 2). The study was conducted according to the Ethics Committees of the National Centre for Tumour Diseases and Hannover Medical School.

### Serological detection of Caspase-3/-7 activity

For serological measurement of Caspase-3/-7 activity, a luminescent substrate based assay was used (Caspase-Glo assay, Promega, Mannheim, Germany) as described previously.^28^

### Explant culture medium and conditions

Collection media consists of Dulbecco's modified Eagle's medium (Gibco, Invitrogen Corp, Carlsbad, CA, USA) supplemented with penicillin (200 IU/l), streptomycin (200 *μ*g/l), Fungizone (2.5 *μ*g/ml), ciprofloxacine (5 *μ*g/ml), and gentamicin (125 *μ*g/ml). Upon plating, all tissues were cultured at 37 °C and 5% CO_2_ until harvest.

### Dissection and plating of explant cultures

Under sterile conditions and 2 h before tissue dissection, 1 cm^3^ haemostatic gelatine dental sponges (Humanus Dental AB) were hydrated in 1 ml collection media at 37 °C in individual wells of a 12-well cell-culture dish. Although sponges were hydrating, tissues were transferred to a 10-cm cell-culture dish with 10 ml collection media for dissection. Samples were then cut into 1 mm^3^ sections using a sterile scalpel blade. Sample sections were washed in fresh collection media. Upon complete hydration of gelatine sponges 1 mm^3^ dissected specimen sections were chosen at random and placed on top of each sponge using sterile forceps. Explant cultures were incubated at 37 °C and 5% CO_2_.

### Treatments of explant cultures

All explants were maintained in collection media for 24 h. At 24 h, media was aspirated carefully to prevent disturbance of plated tissues, and 1 ml of treatment media was then added to the corresponding treatment wells. Treatments were as follows: Untreated, 30 *μ*g 5FU/10 *μ*g Oxali, 30 *μ*g 5FU/10 *μ*g Oxali+200 nM DNLD, 30 *μ*g 5FU/10 *μ*g Oxali+2.5 mM Aspirin, 30 *μ*g 5FU/10 *μ*g Oxali+50 *μ*M celecoxib. Tissues were treated for 96 h. At 96 h, media was aspirated and stored at −20 °C for prostaglandin E2 assay. Following treatment, tissues were processed and FFPE for immunohistochemical analysis.

### Immunohistochemical staining of explant cultures

In all, 5 *μ*m sections were cut using a microtome. Sections were floated onto slides and left to air-dry. Sections were then baked overnight at 65 °C. Samples were deparaffinised by passing through xylene and 100 and 70% ethanol washes. All staining was performed using the DAKO EnVision+ System-HRP (DAB) kit for rabbit (K4010; DAKO) or mouse (K4006; DAKO) primary antibodies. The following primary antibodies were used: ProCaspase-3 (1 : 100; Rabbit polyclonal, HPA002643, Atlas Antibodies), Active Caspase-3 (1 : 200; Rabbit polyclonal, 9661, CST), COX-2 (1 : 400; Mouse monoclonal; CAY160112-1, Cayman (@Cambridge Bioscience)), *β*-Catenin (1 : 300; Neomarkers RB-9035 (Fisher Scientific, Fremont, CA, USA)), Ki-67 (MIB1) (1 : 100; Dako, M7240), ZEB1 (1 : 100; Rabbit polyclonal, HPA027524, Atlas Antibodies). Positive and negative controls were included according to the suppliers′ recommendations (Negative Control Rabbit Immunoglobulin Fraction (Normal), X 0903, Dako; Negative Control Mouse IgG1, X0931, Dako). Following staining, sections were scored semi-quantitatively, on a scale of 0–3 based on the extent and intensity of staining, as described above.

### Double immunofluorescence staining of explant cultures

In all, 5 *μ*m sections were baked, deparaffinised, and hydrated as previously described. Specimens were washed using sodium citrate as antigen retrieval. Cells were permeabilised for 30 min using 0.3% Triton X-100 in 1 × PBS. Sections were then incubated overnight at 4 °C with Rabbit polyclonal Cleaved Caspase-3 Antibody (1 : 200; 9661, Cell Signalling Technology) and Mouse Monoclonal Ki-67 Antibody (MIB1. 1:50; Dako, M7240), diluted in 1 × PBS with 1% BSA and 0.3% Triton X-100. Alexa Fluor 488 Donkey anti-Rabbit IgG (H+L) (A-21206, Life Technologies, Carlsbad, CA, USA) and Alexa Fluor 568 Donkey anti-Mouse IgG (A-10037, Life Technologies) secondary antibodies were used at 1:400 dilutions and incubated for 1 h at room temperature. Nuclei were counterstained with Hoechst stain solution. Slides were mounted using DPX Slide mounting medium (06522, Sigma-Aldrich, St. Louis, MO, USA). As a negative control to ensure specificity of staining, the omission of the primary antibodies from the staining procedure was carried out on some samples. Cells were imaged using a LSM 510 Meta (Carl Zeiss, Jena, Germany) equipped with × 40 oil immersion objective using appropriate excitation, beam splitter, and emission wavelength. Images were processed using ImageJ (1.46r, Wayne Rasband, NIH, USA).

### Apoptosis assay

To analyse apoptosis in each sample sections were processed using the TdT-DAB *in situ* apoptosis detection kit (Trevigen, Gaithersburg, MD, USA, 4810-30-K). Staining was carried out as per manufacturer's instructions, and stainings were scored once again based on the extent and intensity of DAB-positive cells.

### Prostaglandin E2 assay

To measure PGE2 levels according to the treatment used; media was collected from the corresponding samples at 96 h of treatment and was processed using the Prostaglandin E2 Parameter Assay Kit (KGE004B, R&D Systems, St. Cloud, MN, USA). Optical density was determined using a microplate reader set to 450 nm. Evaluation was carried out as per manufacturer's instructions. Standards and controls were included as described in the kit.

### Statistics

Statistical analyses were performed SPSS (IBM, Armonk, NY, USA). Data are given as means±S.E.M. For statistical comparison, ANOVA and subsequent Tukey test were used for normally distributed data. Relations between levels of active Caspase-3 and Ki-67 on CRC TMAs were compared by Spearman correlation test. Serum levels of activated Caspase-3 were compared by using Mann–Whitney's *U* test for non-normally distributed data. *P*-values≤0.05 were considered statistically significant. ROC analyses were performed to calculate the cutoff value of activated Caspase-3 in the serum that correctly predicts treatment failure, that is, PD, at day 14 of CT.

## Figures and Tables

**Table 1:** TMA colon cancer patient clinical characteristics

*N*=93	
Age	
Average	70.06
Range	35–94
Tumour	
T2	6
T3	63
T4	22
Node	
N0	60
N1	20
N2	13
Metastasis	
M0	90
M1	3
Gender	
Male	43
Female	50
Chemotherapy	
Yes	35
No	58
Recurrence	
Yes	22
No	71
Death from disease	
Yes	19
No	74
Patient scoring Caspase-3 levels	
0	44
1	23
2	13
3	13

**Table 2:** Clinical characteristics of the serum colorectal cancer patient cohort

*N*=30	
Gender	
Male	21
Female	9
Disease status	
PR	9
SD	14
PD	7

## Abbreviations

AA: arachidonic acid
ANOVA: analysis of variance
ASCO: American Society of Clinical Oncology
AUC: area under the curve
CAP: College of American Pathologists
CI: confidence interval
CK18: cytokeratin 18
COX-2: cyclooxygenase-2
CRC: colorectal cancer
CT: chemotherapy
DAB: 3,3′-diaminobenzidine
DFS: disease-free survival
FFPE: formalin-fixed paraffin-embedded
5FU: 5-Fluorouracil
EMT: epithelial mesenchymal transition
HCT116: Human Colon Tumour Cell Line
iPLA2: calcium-independent phospholipase A2
LV: leukovorin
MCF7: Breast Cancer Cell Line
ND: neutral density
OS: overall survival
PD: progressive disease
PGE2: prostaglandin E2
PR: partial tumour regression
RECIST: Response Evaluation Criteria in solid tumours
RLU: Relative Light Units
ROC: receiver operating characteristics
SCCHN: squamous cell carcinoma
SD: stable disease
STS: staurosporine
TMAs: tissue microarrays
TUNEL: terminal deoxynucleotidyl transferase dUTP nick end labelling
ZEB: Zinc Finger E-Box Binding Homeobox
