# Supplementary figures and images for: Low levels of Caspase-3 predict favourable response to 5FU-based chemotherapy in advanced colorectal cancer: Caspase-3 inhibition as a therapeutic approach
Source: Cell Death Dis. 2016 Feb 4;7(2):e2087–. doi: 10.1038/cddis.2016.7 (PMC4849164; doi:10.1038/cddis.2016.7)

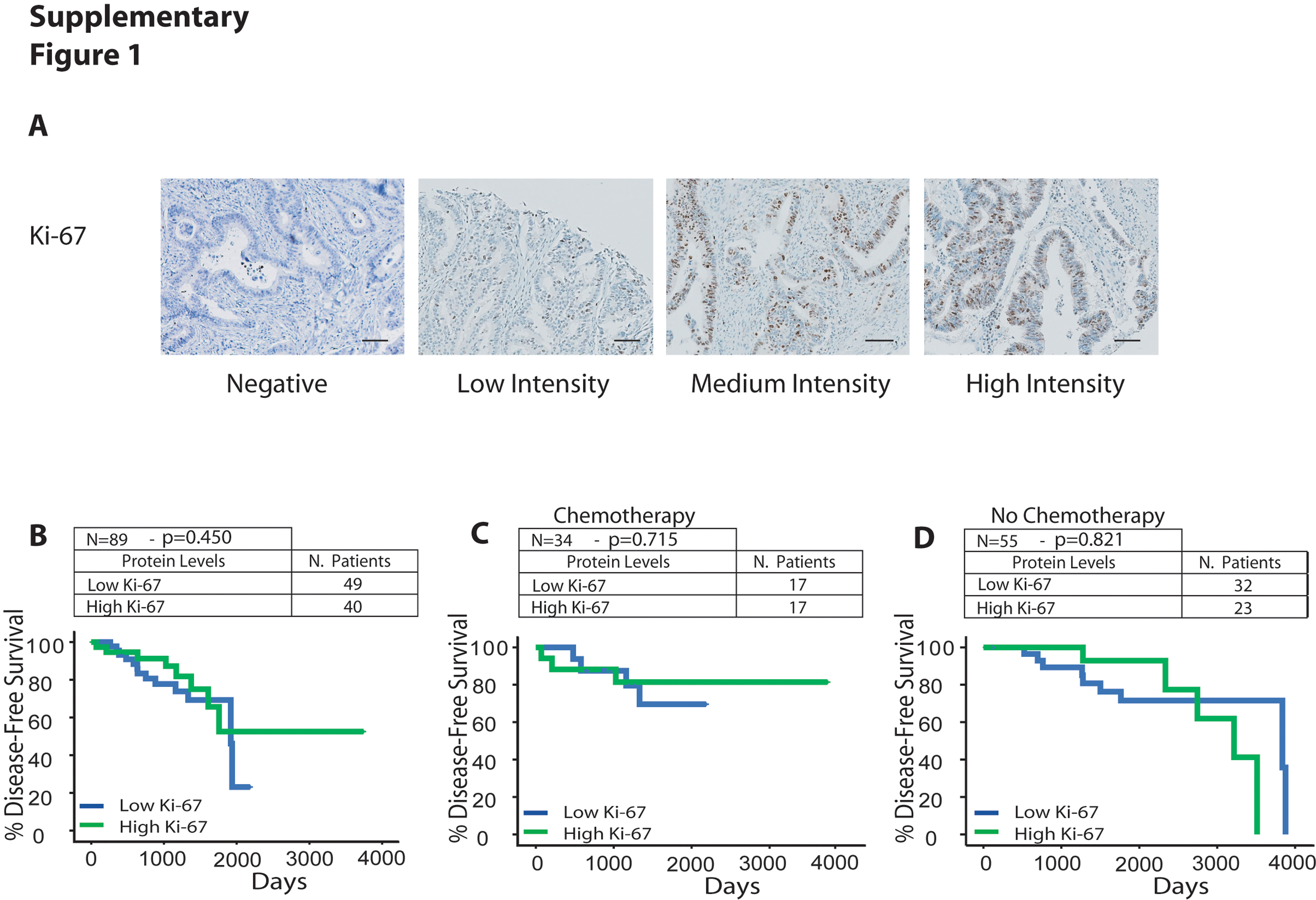

Supplement: Supplementary Figure 1 [file cddis20167x1.tif]
