# Supplementary material for: Low levels of Caspase-3 predict favourable response to 5FU-based chemotherapy in advanced colorectal cancer: Caspase-3 inhibition as a therapeutic approach
Source: Cell Death Dis. 2016 Feb 4;7(2):e2087–. doi: 10.1038/cddis.2016.7 (PMC4849164; doi:10.1038/cddis.2016.7)
Supplement: Supplementary Figure Legends [file cddis20167x2.docx]

**Suppl Figure1. *Expression of active Caspase -3 in stage 2/3 CRC patients is not directly correlated with expression of Ki-67.***

**(A)** TMA sections from a cohort of 89 metastatic CRC patients were stained for active Caspase -3 and Ki-67 and scored based on positive cell structures for the antigens of interest on a scale of 0-3 based on the extent of staining; negative, low, medium or high (Scale bar=100 µm). Ki-67 antigen was confined to the nuclei of epithelial cells of colonic crypt. Ki-67 and active Caspase -3 expression areas did not show overlap which demonstrated no direct correlation between the areas of expression of the protein of interest. Protein levels were compared to patient outcome and patients with low Active Caspase -3 / Ki-67 (blue) did similarly to those with high Active Caspase -3 / Ki-67 (green). **(B)** There was no significant improvement in disease free survival outcome in patients with low levels of Ki-67 when compared to patients with high Ki-67 (p=0.450; N=89, 49 patients expressed low protein levels and 40 patients expressed high levels). **(C)** This non-significant survival pattern was observed whether patients received chemotherapy (N=34, 17 patients expressed low protein levels, with 4 experiencing recurrence, and 17 patients expressed high levels, with 2 experiencing recurrence). **(D)** Patients with low levels of Ki-67 were found to not have a significantly better outcome in terms of disease free survival than patients with high Ki-67 (N=55, 32 patients expressed low protein levels, with 9 experiencing recurrence and 23 patients expressed high levels, with 4 experiencing recurrence).
